# Primary Aortoenteric Fistula: A Rare Case of a Massive Gastrointestinal Bleed

**DOI:** 10.7759/cureus.766

**Published:** 2016-09-01

**Authors:** Simon Ho, Bo Liu, Raul Loya, Ibrahim Koury

**Affiliations:** 1 College of Medicine, University of Central Florida; 2 Diagnostic Radiology, Florida Hospital-Orlando

**Keywords:** aortoenteric fistula, gastrointestinal bleed, abdominal aortic aneurysm

## Abstract

Aortoenteric fistulas (AEFs) are deadly, abnormal connections between the aorta and gastrointestinal (GI) tract. While secondary aortoenteric fistulas (SAEFs) are more common and arise after aortic reconstruction, primary aortoenteric fistulas (PAEFs) are generally caused by abdominal aortic aneurysms (AAAs). PAEFs may present with self-limited GI bleeds called “herald bleeds,” and the fistula often goes undiagnosed until patients undergo laparotomy for a massive GI bleed. We describe a case of a PAEF in a 79-year-old man with known AAA. Due to variable clinical presentations and the rarity of the condition, many patients with PAEF die before an accurate diagnosis is made. In interpreting computed tomography (CT) scans of AEFs, the role of the radiologist is critical in the management of PAEF patients.

## Introduction

Aortoenteric fistulas (AEFs) are deadly, abnormal connections between the aorta and gastrointestinal (GI) tract, first described by Sir Astley Cooper in the early 19th century. While secondary aortoenteric fistulas (SAEFs) are more common and arise after aortic reconstruction, primary aortoenteric fistulas (PAEFs) are generally caused by abdominal aortic aneurysms (AAAs). Rare causes such as syphilis, tuberculosis, peptic ulcer disease, and malignancy have also been described [1]. PAEFs often present with self-limited GI bleeds called “herald bleeds,” and the fistula often goes undiagnosed until patients undergo laparotomy for a massive GI bleed [2]. The incidence of PAEF has been reported to be less than 1% in AAA patients; however, this may be an underestimation due to the asymptomatic nature of AAAs [3]. The overall mortality rate for patients with diagnosed PAEF is 44% [4]. We describe a case of a PAEF in a 79-year-old man with known AAA.

## Case presentation

**Figure 1:**
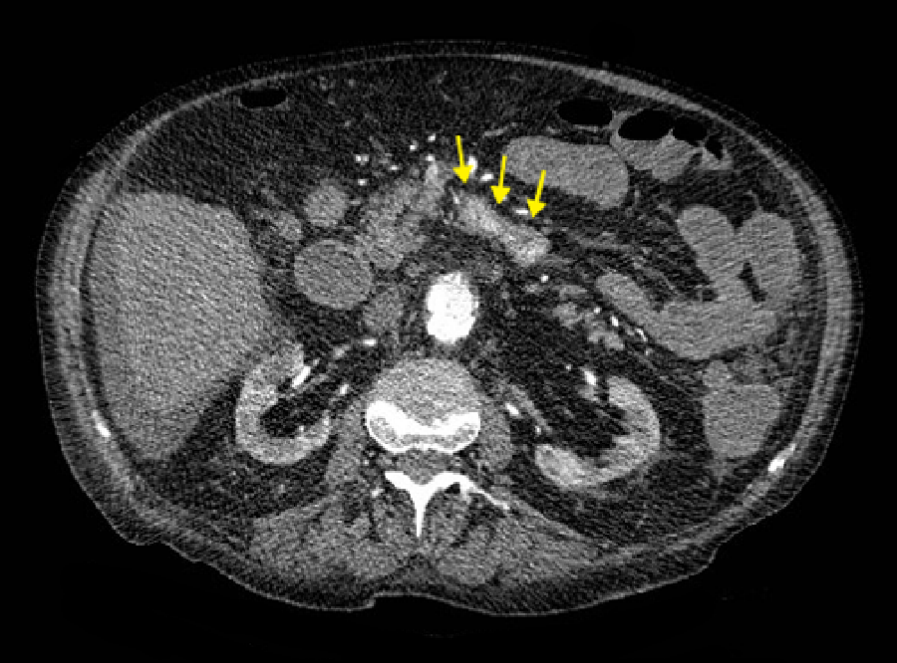
Abnormal Contrast in the Duodenum Axial contrast-enhanced (IV only) CT image at the level of the kidneys showing the abnormal presence of contrast in the 3rd part of the duodenum (arrows).

**Figure 2:**
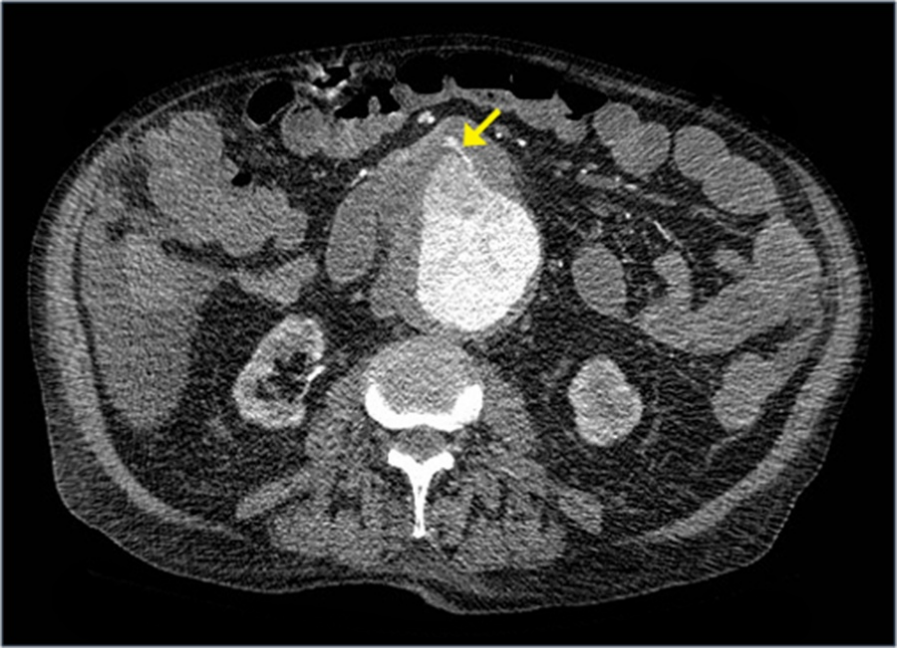
Primary Aortoenteric Fistula (PAEF) Axial contrast-enhanced (IV only) CT image, obtained a few slices inferior to Figure 1, shows an abdominal aortic aneurysm (AAA) measuring up to 11 cm and presence of a fistula leading into the duodenum (arrow).

**Figure 3:**
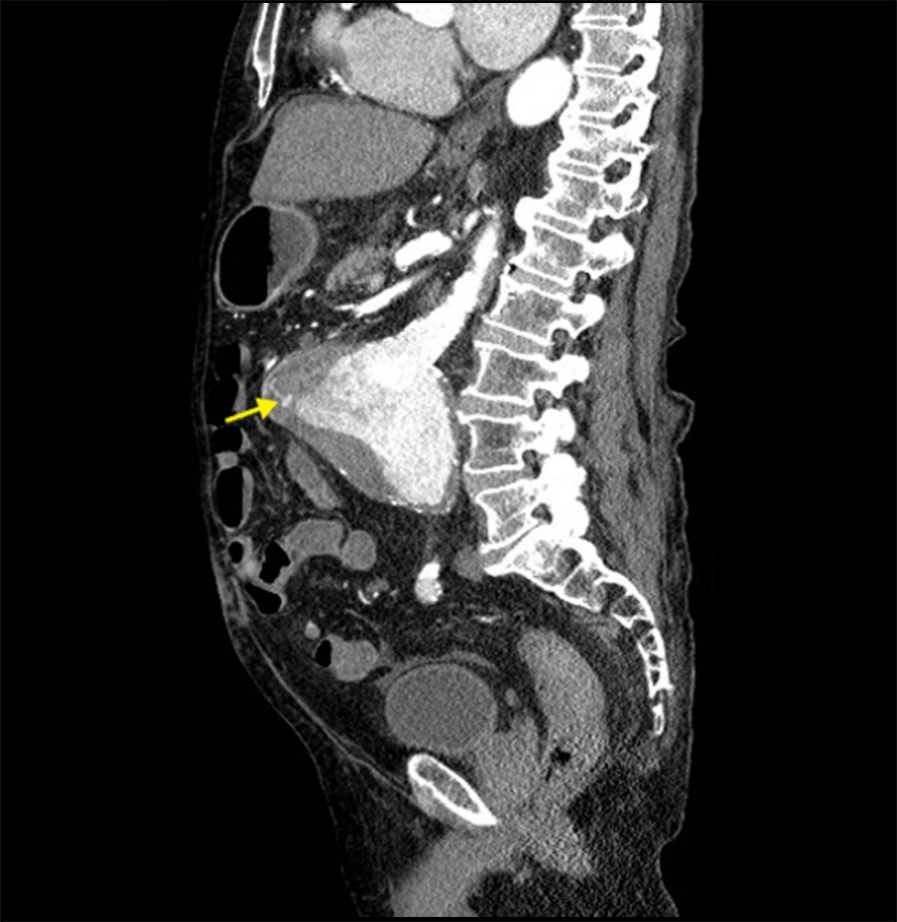
PAEF From Another View Sagittal reformatted contrast-enhanced (IV only) CT image showing the presence of an AAA and associated fistula (arrow).

A 79-year-old man with a past medical history of non-operative AAA, hypertension, and atrial fibrillation was vacationing when he presented to a nearby hospital for both melena and large volume hematemesis. Computed tomography (CT) of the patient’s abdomen showed a saccular 11 cm AAA with fistulization into the third part of the duodenum (Figures 1-3).

**Figure 4:**
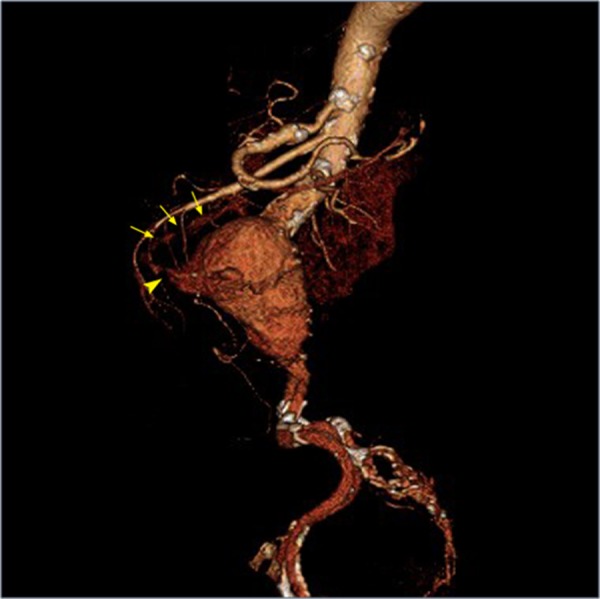
3D Reconstruction of PAEF Left lateral 3D reconstructed contrast-enhanced (IV only) CT image showing the presence of an AAA, fistula (arrowhead), and layering contrast within the 3rd part of the duodenum (arrows).

A 3D reconstruction of the PAEF was also created (Figure 4). The patient was stabilized and transferred to our hospital for emergent repair of the AEF.

The patient was hypotensive with a blood pressure of 81/52 and heart rate of 107. Abdominal palpation demonstrated a pulsatile midline abdominal mass. On his right side, he had impalpable dorsalis pedis pulses, though they were audible via Doppler. Otherwise, his examination had no other positive findings.

The patient was consented for emergent surgery by vascular surgery. A midline laparotomy was performed with dissection down to the aorta. Reconstruction of the aorta using a Dacron graft was completed and augmented with mobilized omentum. Following this, the general surgeon excised the fistulous portion of the duodenum and ligated the remaining ends. An ABthera^TM^ abdominal closure device (Acelity, San Antonio, TX) was placed. The patient was transferred to the intensive care unit (ICU) after hemostasis was obtained.

The patient tolerated the procedure, but he remained hemodynamically unstable secondary to five liters of intraoperative blood loss in addition to prior GI blood loss. He remained in the intensive care unit for eight days post-procedure at which time the health care proxy terminated supportive care.

## Discussion

Diagnosis of a PAEF is often delayed due to its rarity. While 94% of patients present with a GI bleed, the classic triad of a palpable abdominal mass, abdominal pain, and a GI bleed presents only in 11% of individuals [4]. The unreliability of clinical findings accentuates the need for a high index of suspicion for a PAEF in GI bleeding, especially upper GI bleeds.

Patients considered hemodynamically stable are usually evaluated with esophagogastroduodenoscopy (EGD). However, this technique only yields images suggestive of PAEF in a quarter of cases [4]. CT remains the study of choice with the highest detection rate [4], despite a wide range of reported sensitivity (40-94%) [5-6]. Imaging features suggestive of PAEF include air within the aortic wall, focal bowel wall thickening, disruption of pre-aortic fat, or contrast in proximity or in the bowel. Other imaging modalities, such as angiography and ultrasound, only match the sensitivity of EGD at best [4].

## Conclusions

CT, like other imaging modalities, has increased in overall use and detection rate as a diagnostic tool for PAEF. While EGD remains the necessary first-line investigation, CT is the best investigative tool if AEF is suspected. This is likely due to CT’s ability to detect indirect signs better than other modalities. Surgical technique has also been improving; at present, mortality associated with PAEF repair is 34% compared with 44% prior to 1994 [4]. Due to variable clinical presentations and rarity of the condition, many patients with AEF die before an accurate diagnosis is made. Outcomes depend on the promptness of the diagnosis and time until surgery [7]. Therefore, the role of the radiologist is critical in the management of PAEF patients.
